# Vaccination with the Crimean-Congo hemorrhagic fever virus viral replicon vaccine induces NP-based T-cell activation and antibodies possessing Fc-mediated effector functions

**DOI:** 10.3389/fcimb.2023.1233148

**Published:** 2023-08-21

**Authors:** F. E. M. Scholte, E. Karaaslan, T. J. O’Neal, T. E. Sorvillo, S. C. Genzer, S. R. Welch, J. D. Coleman-McCray, J. R. Spengler, M. H. Kainulainen, J. M. Montgomery, S. D. Pegan, E. Bergeron, C. F. Spiropoulou

**Affiliations:** ^1^ Viral Special Pathogens Branch, Division of High-Consequence Pathogens & Pathology, Centers for Disease Control & Prevention, Atlanta, GA, United States; ^2^ Division of Biomedical Sciences, School of Medicine, University of California, Riverside, Riverside, CA, United States

**Keywords:** CCHFV, vaccine, antibody, Fc-mediated effector function, nucleoprotein

## Abstract

Crimean-Congo hemorrhagic fever virus (CCHFV; family *Nairoviridae*) is a tick-borne pathogen that frequently causes lethal disease in humans. CCHFV has a wide geographic distribution, and cases have been reported in Africa, Asia, the Middle East, and Europe. Availability of a safe and efficacious vaccine is critical for restricting outbreaks and preventing disease in endemic countries. We previously developed a virus-like replicon particle (VRP) vaccine that provides complete protection against homologous and heterologous lethal CCHFV challenge in mice after a single dose. However, the immune responses induced by this vaccine are not well characterized, and correlates of protection remain unknown. Here we comprehensively characterized the kinetics of cell-mediated and humoral immune responses in VRP-vaccinated mice, and demonstrate that they predominantly target the nucleoprotein (NP). NP antibodies are not associated with protection through neutralizing activity, but VRP vaccination results in NP antibodies possessing Fc-mediated antibody effector functions, such as complement activation (ADCD) and antibody-mediated cellular phagocytosis (ADCP). This suggests that Fc-mediated effector functions may contribute to this vaccine’s efficacy.

## Introduction

Crimean-Congo hemorrhagic fever virus (CCHFV) is a tick-borne pathogen (*Nairoviridae*; *Bunyavirales*), with a broad geographic distribution. While mostly causing asymptomatic infection in a wide range of animals, CCHFV can cause severe disease in humans. Human cases are reported in various countries in Europe, Asia, and parts of Africa. Licensed vaccines and specific, effective antivirals remain unavailable, but are urgently needed to curb and prevent outbreaks.

CCHFV has a negative-sense, tri-segmented genome. The large (L) segment encodes the RNA-dependent RNA polymerase. The medium (M) segment encodes the glycoprotein precursor (GPC), which is further processed into various glycoproteins including Gn and Gc required for attachment and entry of viral particles, as well as non-structural glycoproteins, including GP38. The small (S) segment encodes the nucleoprotein (NP). As observed for other bunyaviruses, NP is abundant and highly immunogenic, and is often used as a target in diagnostic assays.

We previously developed a single-dose virus-like replicon particle (VRP) vaccine that provides complete protection against homologous and heterologous lethal CCHFV challenge in mice (Scholte et al., 2019; Spengler et al., 2019; Spengler et al., 2021). VRPs contain the CCHFV S and L genome segments, while the glycoproteins normally encoded by the M segment are provided *in trans*, resulting in particles with single-round replication, and authentic transcriptional and translational activity of 2 of the 3 genome segments. Vaccines containing replicating RNA can drive high levels of intracellular protein expression and can induce both humoral and cell-mediated immunity, mimicking authentic virus infection. The VRP vaccine platform combines the immunogenicity of replicating RNA with authentic entry (delivery) in host cells through utilization of the viral glycoproteins, and relieves the need for adjuvant or other delivery vehicles.

The VRP vaccine provides protection from disease after a single dose when given as little as 7 days before challenge, while vaccination 3 days before CCHFV challenge protects mice from death but not from disease (Spengler et al., 2021). Despite the demonstrated efficacy of this vaccine, the vaccine-induced immune responses contributing to protection remain largely uncharacterized, and correlates of protection are unknown. VRP vaccination results in the production of NP and L polymerase, but not the glycoproteins. An effective CCFV vaccine will likely require induction of both B and T cell responses (Hinkula et al., 2017; Leventhal et al., 2022).

In this study, we characterized the kinetics of humoral and cellular immune responses in mice after CCHF VRP vaccination. Mice were vaccinated with a single dose of the VRP vaccine and euthanized at 8 timepoints (range: 1-56 days) after vaccination. We analyzed innate, cell-mediated and humoral immune responses, including Fc-mediated antibody effector functions. Vaccinated animals developed a strong IgG response against NP, while antibodies against the glycoproteins (Gc, Gn, GP38) were not detected. NP-specific antibodies possessed antibody-dependent complement deposition (ADCD) and antibody-dependent cellular phagocytosis (ADCP) activity. Cellular immune responses after VRP vaccination included NP-specific T cell activation, resulting in IFN-*γ* production. Early innate immune responses after vaccination or overt sex-specific differences were not detected. Taken together, our data suggest that NP-specific immunity is key for the efficacy of the VRP vaccine.

## Methods

### Cells

Vero-E6 cells stably expressing codon-optimized CCHFV Oman strain glycoprotein were generated by cloning the ORF into an episomal vector (System Biosciences) with an additional puromycin resistance cassette, transfecting cells with this construct, and selecting stable clones under puromycin selection. Uniform expression of the glycoprotein was verified by immunostaining with monoclonal antibodies 11E7 and 13G8 (BEI Resources). Sequence of the transgene carried by the cell line was verified by PCR with Phusion Human Specimen Direct PCR Kit and next-generation sequencing (Illumina). Cells were cultured in DMEM supplemented with fetal calf serum, non-essential amino acids, sodium pyruvate, L-glutamine, antibiotics, and puromycin. THP-1 cells (ATCC TIB-202) were cultured in RPMI supplemented with fetal calf serum, and antibiotics.

### VRP vaccine

The CCHFV VRP vaccine contains the S and L genome segments of CCHFV strain IbAr10200 combined with ectopically expressed Oman strain GPC (Scholte et al., 2019; Spengler et al., 2019; Spengler et al., 2021). The stock used in this study also expresses the ZsGreen reporter gene from the S segment ORF, using a similar strategy as described for infectious recombinant CCHFV (Welch et al., 2017; Welch et al., 2019). The original VRP stock was generated by transfecting Huh7 cells with plasmids expressing the IbAr10200 S and L genome segments under control of a T7 promoter, as well as plasmids encoding T7, NP, and human codon-optimized GPC and L proteins. Stocks were amplified and quantified by TCID_50_ determination in Vero-E6 cells stably expressing the codon-optimized Oman GPC. The VRP sequence was verified by Illumina next-generation sequencing and confirmed to be mycoplasma free.

### Animals

Six-week-old male and female C57BL6/J mice (Jackson Laboratory #000664) were kept in a climate-controlled laboratory with a 12 h day/night cycle; provided commercially available mouse chow (Teklad Global 18% Protein Rodent Diet) and water *ad libitum*; and group-housed on autoclaved corn cob bedding (Bed-o’Cobs^®^ ¼”, Anderson Lab Bedding) with cotton nestlets in an isolator-caging system (Tecniplast GM500) with a HEPA-filtered inlet and exhaust air supply. Mice were vaccinated subcutaneously with a target dose of 1 x 10^5^ TCID_50_ VRP in the dorsum/interscapular region (back-titer dose: 8 x 10^4^ TCID_50_). Control animals were mock-vaccinated with DMEM only. Equal numbers of male and female mice were divided between 8 groups representing the pre-determined terminal sampling timepoints. At each predetermined timepoint, 14 mice were euthanized (5 male and 5 female vaccinated animals; 2 male and 2 female mock-vaccinated mice). At the time of euthanasia, terminal blood was collected by intracardiac puncture under deep isoflurane anesthesia in lithium heparin microtubes (Sarstedt). Animals were subsequently euthanized and tissues were harvested at necropsy. All animal experiments were approved by the CDC Institutional Animal Care and Use Committee and performed in an AAALAC-International approved facility.

### RNA extraction and detection

RNA was extracted from lithium-heparinized whole blood and homogenized tissue samples using the MagMAX-96 Total RNA Isolation Kit (ThermoFisher) on a 96-well ABI MagMAX extraction platform with a DNaseI treatment step. RNA was quantified using a one-step, real-time reverse-transcription polymerase chain reaction (RT-PCR) assay targeting the NP gene sequence (Spengler et al., 2019) and normalized to 18S with a SuperScript III Platinum One-Step qRT-PCR Kit. Relative copy numbers were determined based on a dilution series of an *in vitro* RNA transcript of known copy number run in parallel. Relative RNA levels of selected immune genes in blood and lymph node tissues were quantified using murine Taqman assays (ThermoFisher) and normalized to β-actin mRNA levels.

### Luminex cytokine detection

Plasma samples were analyzed using a magnetic Th1/Th2 cytokine 11-plex mouse ProcartaPlex panel (Luminex EPX110-20820-901) per manufacturer’s instructions.

### Protein expression and purification

Kosovo Hoti strain sequences were used for the expression of all proteins. The NP sequence was optimized for bacterial expression and cloned into pET28a (Twist Bioscience). After transformation into *E. coli* BL21 (DE3) strain, a bacterial culture was grown, and induced with 1 mM IPTG when OD was between 0.4-0.6, and transferred to 16°C for overnight incubation. Cells were harvested by centrifugation, resuspended in lysis buffer (500 mM NaCl, 20 mM Tris-Cl [pH 7], 0.1% Triton-X, 5% glycerol, 1 mM MgCl_2_, 25 U/ml benzonase), and sonicated. The cleared lysates were filtered through 0.2-micron PES membranes and loaded onto HisTrap Excel columns (Cytiva) for immobilized metal affinity chromatography. Following His purification, N-terminal His-GST was cleaved with HRV-3C protease cleavage enzyme (3CC-N3133, Acro Biosystems).

The CCHFV Gn, Gc, and GP38 sequences were cloned into pTWIST (for Gn) or pEEV (Kainulainen et al., 2021) (for Gc and GP38) plasmids by Twist Bioscience. Proteins were expressed in Expi293F cells after transfection using FectoPro transfection reagent (Polypus). For the constructs with furin cleavage sites, plasmids were co-transfected with furin plasmid at a 4:1 ratio. Supernatants were harvested 4-6 days post transfection, filtered through 0.2-micron PES membranes and purified by IMAC using HisTrap Excel columns. Proteins were further purified using size exclusion chromatography, quantified, and stored at -80°C. All expression and purification steps were confirmed by polyacrylamide gel electrophoresis. For additional details, see **supplemental Methods**.

### ELISA

Plates were coated with antigen in PBS and incubated overnight at 4°C. Wells were washed with PBS-T (0.1% Tween-20 in PBS) and blocked with blocking buffer (5% [w/v] non-fat dry milk in PBS-T). Plasma samples diluted in blocking buffer were added to the wells at 1:1000 (IgG) or 1:500 dilutions (IgM). After 1h incubation at RT, wells were washed and anti-mouse IgG or IgM HRP was added to the wells, and incubated 1h at RT. After washing, TMB Ultra ELISA substrate was added and incubated 10 min at RT. The reaction was stopped using ELISA stop solution and OD was read at 450 nm. Antibody activity units were determined based on calibrator curves generated using mouse IgG and IgM standards. Antibody concentrations in samples were determined by interpolating the concentrations of the standards that corresponded to the absorbance value of the sample. For additional details, see **Supplemental Methods**.

IgG avidity of NP-specific antibodies was determined by treating plasma samples with 1M NH_4_SCN prior to ELISA analysis as described above. IgG avidity index was calculated by dividing the mean OD of NH_4_SCN-treated plasma by the mean OD of PBS-treated plasma, and multiplying this number by 100 to obtain a percentage.

### Flow analysis

Cells were incubated with mouse Fc block and surface stains (**Supplementary Table S1**) for 30 min on ice. Cell viability was determined using Tonbo Ghost Dye Violet 510. Cells were washed and resuspended in Cytofix/Cytoperm (BD) for 20 min on ice. Stained cells were analyzed on a Stratedigm S1000EXi. For additional details, see **Supplemental Methods**.

### IFN-*γ* ELIspot

Splenocytes were diluted to 2 x 10^6^ cells/ml in RPMI containing 10% heat-inactivated FBS and antibiotics, and seeded onto ELIspot plates (MabTech 3321-4HPW) containing peptides spanning the CCHFV IbAr10200 NP protein (AbClonal, 15-mers with 9 AA overlap). Alternatively, peptides spanning the NSm-Gc domain of the Oman GPC were used (Genscript; 15-mers with 4 AA overlap). PMA and DMSO were used as positive and negative controls, respectively. Spots were counted using a CTL ELIspot reader and normalized to PMA-induced spots.

### Antibody-dependent complement deposition and antibody-dependent cellular phagocytosis

These assays were adapted from (Butler et al., 2019; Fischinger et al., 2019). Recombinant NP was biotinylated (ThermoFisher 21435) and coupled to fluorescent red or green neutravidin microspheres (ThermoFisher F8775/F8776). Antigen-coated beads were incubated with mouse plasma (2 h at 37°C). For ADCD assays, guinea pig complement (Cedarlane CL4051) diluted in gelatin veronal buffer (CompTech B102) was added and incubated 15 min at 37°C. Beads were washed and incubated 15 min at RT with FITC-conjugated anti-guinea pig complement C3 (MP Biomedicals 0855385). For ADCP assays, beads were incubated overnight at 37°C with 1 x 10^4^ THP-1 cells per well. Beads or cells were analyzed on a Guava cytometer. Fold ADCD activation was calculated using naïve mouse plasma. Phagocytic score was calculated by multiplying the percentage of bead-positive cells with the overall median fluorescence intensity. For additional details, see **Supplemental Methods**.

### Statistics and data visualization

**Figure 1:**
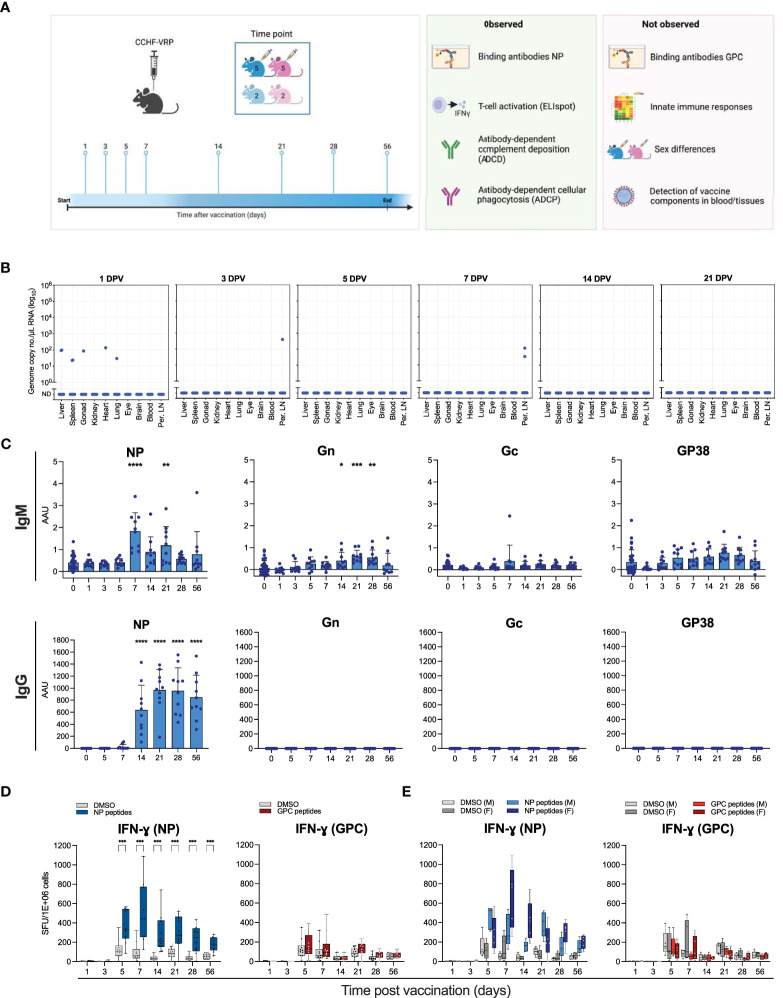
Humoral and cellular immune responses to CCHFV VRP vaccination. **(A)** Schematic study overview. Male and female C57BL6/J mice were divided into 8 experimental groups containing 10 vaccinated animals and 4 controls, sexes distributed evenly. Animals were given a single dose of 1 x 10^5^ TCID_50_ CCHFV VRP particles. At the indicated timepoints after vaccination (days post vaccination; DPV), 14 animals were euthanized (10 vaccinated and 4 control animals) and samples were collected. **(B)** VRP-derived CCHFV S segment RNA levels detected by RT-qPCR in various tissues at the indicated timepoints post vaccination. **(C)** IgG and IgM antibodies against CCHFV NP, Gn, Gc, and GP38 were quantified by ELISA using the corresponding purified proteins. **(D)** Splenocytes were isolated from vaccinated and control C57BL6/J mice and IFN-ɣ production was measured by ELIspot. Splenocytes were incubated with peptides spanning NP or NSm-Gc (GPC). Spot counts were normalized to PMA-induced spots. **(E)** Data represented in panel D was reformatted to show male (M) v. female (F) mice. Dots represent individual animals. Means and standard deviations are indicated. DPV, days post vaccination; Per. LN, Peripheral lymph nodes; AAU, arbitrary antibody units; SFU, spot-forming units. Statistical significance: *p<0.05, **p<0.01, ***p<0.001, ****p<0.0001. If not indicated, no significance was found.

Data were visualized and analyzed for statistical relevance using GraphPad Prism v.9. **Figure 1A** was created with Biorender.com.

## Results

### VRP RNA is transiently detected in tissues up to 7 days post vaccination and does not induce detectable innate immune responses

To characterize the kinetics of cellular and humoral immune responses that potentially correlate with protection induced by the CCHFV VRP vaccine in immunocompetent mice, a cohort of C57BL6/J mice was vaccinated with a single dose (1 x 10^5^/mouse) of the VRP vaccine. Animals were divided into 8 experimental groups containing 10 vaccinated animals and 4 mock-vaccinated controls, with equal numbers of males and females (**Figure 1A**). A subset of animals was euthanized at pre-determined timepoints, ranging 1-56 days post vaccination. Blood, liver, spleen, peripheral lymph nodes, and a selection of other tissues were collected.

To investigate if RNA derived from the VRP vaccine could be detected after vaccination, RNA was isolated from various tissues, including blood, lymph nodes, and spleen, and analyzed for the presence of CCHFV S segment RNA by RT-qPCR. One day after vaccination, low levels of VRP-derived RNA could be detected in the liver, spleen, gonads, heart and lungs of one animal, and in the liver and spleen of a second animal. No VRP-derived RNA could be detected in any of the tested tissues of the other 8 vaccinated animals 1 day after vaccination. In addition, low levels of VRP-derived RNA could be detected in the peripheral lymph nodes of 1 animal 3 days post vaccination, and in the peripheral lymph nodes of 2 animals 7 days post vaccination (**Figure 1B**).

Additionally, we looked at vaccine-derived protein expression by flow cytometry. In this study, the CCHF VRP vaccine expressed a reporter gene (ZsGreen1; ZsG) from the S segment. This allows direct visualization of cells containing VRP RNA, as ZsG is expressed alongside NP. To assess the presence of ZsG-positive cells as an indicator of VRP-derived RNA, liver, spleen and peripheral lymph node samples were processed for flow analysis 1 and 3 days post vaccination. No ZsG signal was detected in any of these tissues harvested at either timepoint (data not shown).

Next, we examined the induction of localized or systemic innate immune responses after vaccination. It was previously observed that the VRP vaccine protects mice from lethal CCHFV challenge given 3 days after vaccination (Spengler et al., 2021). This could potentially be due to innate immune responses induced by VRP vaccination. The replicating VRP RNA may be recognized by pattern recognition receptors and result in the production of IFNs and proinflammatory cytokines. The presence of VRP-derived antigens, such as the glycoproteins studding the particles and newly synthesized NP and L, can also induce innate immune responses (Kang and Compans, 2009). Generally, immune cells, including dendritic cells and monocytes/macrophages at the vaccination site or draining lymph nodes take up the vaccine and express cytokines upon activation, such as IFN-ɣ, IFN-α, and IL-6. This activation ultimately results in efficient induction of the adaptive immune response (Matsumura et al., 2022). To assess if the VRP vaccine induces innate immune responses, we quantified levels of cytokines involved in the T helper cell response in plasma samples obtained during the first 5 days after vaccination. We did not observe any significant changes in cytokine levels (**Supplementary Figure S1A**).

In addition, we examined the expression levels of a limited selection of immune genes (ISG15, CCL-2, IFN-β, and IFN-ɣ) by RT-qPCR using RNA extracted from blood and lymph node samples obtained during the first week after vaccination. No upregulation of these genes was observed (**Supplementary Figure S1B**).

### VRP vaccination induces humoral and cellular immune responses predominantly targeting NP

We previously found that CCHFV VRP vaccination protects mice from lethal infection. In these mice, NP-specific antibodies were found in samples collected pre-challenge at 28 days post vaccination (Scholte et al., 2019; Spengler et al., 2019). Additionally, we found that mice vaccinated 3 days before lethal CCHFV challenge are protected from death (Spengler et al., 2021). However, the longitudinal kinetics of vaccine-induced humoral and cellular immunity associated with these protective vaccination timepoints have not been examined. To fully characterize the kinetics of CCHFV-specific antibody development following VRP vaccination in immune-competent mice, we analyzed plasma samples obtained at the indicated timepoints (1-56 days post vaccination). IgM and IgG binding antibodies against CCHFV NP, Gn, Gc and GP38 were analyzed by ELISA (**Figure 1C**). Similar to previous reports, we detected robust antibody levels against NP at 28 days post vaccination. We detected NP-specific IgM and IgG as early as 7 days post vaccination, with IgG levels reaching a plateau on day 21, and IgM levels waning after day 7. Antibodies against any of the GPC components (Gc, Gn, GP38) could not be detected. This study expands our understanding of the humoral response to CCHFV VRP vaccination with additional timepoints providing insight into the antibody kinetics, as well as 2 novel antigens (Gn and GP38). No differences in antibody responses were observed between male and female animals (data not shown).

In previous studies, the cellular immune response to VRP vaccination was not assessed. Recent studies suggest that both cellular and humoral immune responses are required for vaccine-mediated protection against viral infection. To assess the cellular immune response to CCHFV VRP vaccination, we used ELIspot assays to examine antigen-specific T cell activation. Splenocytes were collected from vaccinated and mock-vaccinated control animals at the indicated timepoints and restimulated with CCHFV NP peptides. A peak in NP-specific T cell activation was observed 7 days post vaccination (**Figure 1D**). Male animals had a slightly earlier response (5 days after vaccination), whereas female mice overall showed a stronger response to NP restimulation (**Figure 1E**). Restimulation of splenocytes with CCHFV GPC (NSm-Gc) peptides yielded a modest response 5 days post vaccination, which waned quickly after. Female vaccinated animals had a slightly stronger response to the GPC peptides than male animals (**Figure 1E**). Overall, the antigen-specific T-cell activation after VRP vaccination was predominantly NP-based, and only weak GPC-specific activation could be detected.

**Figure 2:**
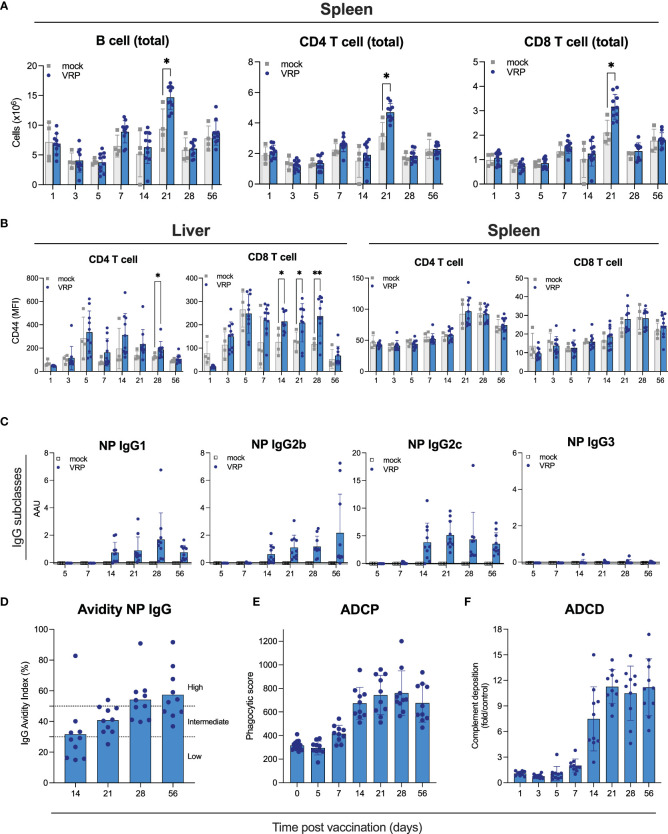
Cellular immune responses and Fc-mediated antibody functionality induced by VRP vaccination. **(A, B)** Immune cells were isolated from spleen, peripheral lymph nodes, and liver of vaccinated and control C57BL6/J mice at the indicated timepoints post vaccination. Quantity **(A)** and activation status **(B)** of B cells, CD4^+^ and CD8^+^ T cells was determined by flow cytometry. **(C)** Isotypes of anti-NP IgG antibodies were determined by ELISA isotype-specific secondary antibodies. **(D)** Avidity of IgG antibodies against CCHFV NP was determined by treating plasma samples with 1 M NH_4_SCN prior to ELISA analysis. IgG avidity index was calculated by dividing the mean OD of NH_4_SCN-treated plasma by the mean OD of PBS-treated plasma. **(E)** Antibody-dependent cellular phagocytosis (ADCP) activity of NP-specific antibodies induced by VRP vaccination was quantified by incubating plasma of vaccinated or control mice with CCHFV NP-coated green fluorescent beads. After formation of immune complexes, THP-1 cells were added and incubated overnight. The next day, cells were washed and analyzed on a Guava cytometer. Phagocytic score was calculated by multiplying the percentage of bead-positive cells with the overall median fluorescence intensity. **(F)** Antibody-dependent complement deposition (ADCD) activity of NP-specific antibodies induced by VRP vaccination was determined by incubating plasma of vaccinated or control mice with CCHFV NP-coated red fluorescent beads. After formation of immune complexes, guinea pig complement was added, followed by the addition of a C3-specific FITC-labeled antibody. ADCD activity was calculated as fold induction over mock-vaccinated animals. Dots represent individual animals. Means and standard deviations are indicated. Statistical significance: *p<0.05, **p<0.01. If not indicated, no significance was found.

To further examine effects of vaccination on immune cells, we assessed B and T cells in liver, spleen, and peripheral lymph nodes at the indicated timepoints after vaccination by flow analysis (**Figures 2A, B**; **Supplementary Figure S2**). Liver and spleen are primary targets of CCHFV, and peripheral lymph nodes were selected as potential initial sites of VRP replication, VRP-induced antigen presentation, and immune cell activation. We determined total numbers of B cells, and CD4^+^ and CD8^+^ T cells, as well as T cell activation status (CD69^+^, CD44^+^). Overall, few vaccine-specific changes were observed at any of the tested timepoints. We saw a modest [but statistically significant (p<0.01)] increase in total B cells, and CD4^+^ and CD8^+^ T cells in the spleens of vaccinated animals 21 days post vaccination, compared to mock-vaccinated animals euthanized on the same day (**Figure 2A**). In the liver, we found elevated levels of CD4^+^ CD44^+^ and CD8^+^ CD44^+^ T cells at various timepoints (14, 21, 28 days) after vaccination (**Figure 2B**). No differences were seen between vaccinated and control animals in the lymph node samples (**Supplementary Figure S2**).

### NP-specific antibodies possess Fc-mediated antibody functionality

Both neutralizing and non-neutralizing antibodies can contribute to protection against CCHFV infection. Antibodies targeting NP have been reported to lack neutralizing activity (Zivcec et al., 2017; Leventhal et al., 2022; Hu et al., 2023). Non-neutralizing antibodies can be protective through other mechanisms, including Fc-mediated effector functions like antibody-dependent cellular cytotoxicity (ADCC), antibody-dependent cellular phagocytosis (ADCP) and antibody-dependent complement deposition (ADCD). We set out to characterize the vaccine-induced NP-specific antibodies and elucidate how their non-neutralizing activity could contribute to protection. First, we determined the IgG subclass profiles of NP-specific antibodies, as this can provide some insight into antibody functionality. C57BL6/J mice can produce IgG1, IgG2b, IgG2c, and IgG3. Murine IgG1 antibodies poorly facilitate Fc-mediated effector functions, whereas IgG2b and IgG2c have been described to facilitate Fc-mediated effector functions (Collins, 2016). We detected NP-specific IgG1, IgG2b, and IgG2c, but not IgG3 (**Figure 2C**), indicating that these NP-specific antibodies have the potential for Fc-mediated effector functionality. Next, we determined avidity of IgG NP antibodies produced in response to vaccination. Avidity is a measure of the strength of binding between IgG and its epitope. Avidity of NP IgG increased gradually over time, indicating antibody maturation, enhancing their protective activity (**Figure 2D**).

To investigate the potential mechanisms underlying the expected contribution of the NP-based humoral response to vaccine-mediated protection, we measured Fc-mediated antibody functionality (**Figures 2E, F**). Vaccine-induced NP-specific antibodies possessed both ADCD and ADCD activities. ADCD activity could first be detected 7 days after vaccination and plateaued 28 days after vaccination, largely following NP IgG kinetics. ADCP activity associated with NP-specific plasma antibodies followed similar kinetics. These results support a protective role for VRP-induced NP antibodies through Fc-mediated effector functions.

## Discussion

We previously reported a single-dose CCHFV VRP vaccine that provides complete protection against lethal infection in mice. However, protective immune responses induced by this vaccine are not well characterized, and the correlates of protection remain unknown. In this study, we longitudinally characterized the humoral and cellular immune responses to VRP vaccination in C57BL6/J mice. We demonstrate that vaccination induced a strong NP-based humoral response, and that these anti-NP antibodies possess Fc-mediated effector functions (ADCP and ADCD). To the best of our knowledge, antibodies possessing Fc-mediated effector functions have not been described yet for CCHFV vaccines or in CCHFV-infected animals or human patients. These assays can provide novel insights into the underlying mechanisms of protection by non-neutralizing antibodies.

The correlates of natural or vaccine-mediated protection against CCHFV infection remain unknown. Various studies suggest that both humoral and cell-mediated immunity contribute to protection (Goedhals et al., 2017; Hinkula et al., 2017; Hawman et al., 2021; Hawman et al., 2023; Leventhal et al., 2022). Neutralizing antibodies targeting Gc were found in human CCHF survivors, along with non-neutralizing NP- and GP38-reactive antibodies. Earlier studies have also suggested that CCHFV neutralizing antibodies are not required for protection (Hinkula et al., 2017; Haddock et al., 2018; Zivcec et al., 2018; Hawman et al., 2019; Hawman et al., 2020; Appelberg et al., 2021; Hawman et al., 2021).

Several vaccine candidates for CCHFV have been reported, some of which target the GPC whereas others are NP-based. Both antigens are able to provide protection, though the efficacy appears to be dependent on the vaccine platform used. GPC-based vaccines are more likely to induce neutralizing antibodies, while it is unclear whether cell-mediated and/or humoral immune responses are responsible for the protective efficacy of NP-based vaccines. Antibodies targeting CCHFV NP have been reported to lack neutralizing activity (Zivcec et al., 2017; Mears et al., 2022). NP is an internal viral protein involved in packaging viral genomes and not generally exposed on the viral surface, leading to the assumption that NP-mediated protection may be predominantly T-cell based. Alternatively, non-neutralizing antibody effector functions may contribute to protection. During natural infection, CCHFV NP is abundant, immunogenic, and as the predominant antigen often used in diagnostic assays. Human CCHF survivors produce NP antibodies, and NP-specific T cell responses have been identified in human survivors (Goedhals et al., 2017). While CCHFV strains can be genetically diverse, the NP protein is highly conserved, supporting its use as a vaccine target over the more genetically variable GPC. Various vaccines using NP as the sole target have been described for other viral pathogens, including Ebola virus, hantavirus, influenza virus, Lassa virus, and Rift Valley fever virus (Clegg and Lloyd, 1987; Xu et al., 1992; Wilson and Hart, 2001; Epstein et al., 2005; Boshra et al., 2011; Tsuda et al., 2011). CCHFV vaccine candidates using NP as the target include those based on adenovirus vector, modified *Vaccinia ankara* virus vector, and alphavirus replicon-based platform, with protective efficacy ranging from 0-100% (Dowall et al., 2015; Zivcec et al., 2018; Leventhal et al., 2022).

Antibodies play an essential role in host defense by recognizing pathogens and infected cells. Although neutralizing antibodies are often seen as a key mechanism of protection against many viral pathogens, antibodies are able to mediate additional immune functions contributing to vaccine-elicited protection as well as post-infection control and resolution of disease. Besides preventing pathogen entry into host cells by directly binding to pathogens (neutralization), antibodies can provide protection by promoting clearance of pathogens *via* interactions with both the innate and adaptive immune system, engaging a range of antimicrobial processes. Antibodies and antigens form immune complexes resulting in sequestration and uptake of pathogens, elimination of infected cells, increased antigen presentation and regulate inflammation regulation. These diverse antibody effector functions are regulated by differential modifications of the Fc domain, which instructs the immune system downstream and ultimately results in lysis or phagocytosis of viral particles or infected cells.

Several studies have suggested that antibody effector functions besides neutralization are important for providing protection from lethal CCHFV infection. The GP38-targeting non-neutralizing monoclonal antibody was reported to protect against lethal challenge (90%) in a complement-dependent manner (Golden et al., 2019). The CCHFV-specific antibodies induced in this study and others (Leventhal et al., 2022) are predominantly of the IgG2b/c subclasses, which possess potent Fc-effector activity, supporting the notion that Fc-mediated effector functions are involved in protection from lethal CCHFV. Similar observations have been made for Ebola virus immunity: an Ebola virus GP neutralizing antibody without Fc effector functions provided modest protection in a lethal mouse model (30%). However, functional Fc variants were protective *in vivo*, suggesting that Fc effector functions can play a crucial role in antiviral protection (Gunn et al., 2021).

VRP vaccination induced NP-specific T cell activation. Whether this contributes to protection cannot be ascertained without studies involving knock-out animal models or passive transfers of antibodies or T cells. It is conceivable that T cell responses contribute to protection when challenged 3 days after vaccination, given the detection of NP-specific T cell activation 5 days after vaccination. Humoral responses may also contribute to this early protection. While we did not detect any changes in the examined innate immune components, we cannot rule out the involvement of the innate immune response. The induction of innate immune responses facilitates the development of adaptive immune responses, and vaccination at 3 days before challenge could therefore enhance the development of adaptive immunity *via* this mechanism.

When assessed by flow cytometry, few changes could be detected in total cell count or activation state of B and T cells, in spleen, liver and peripheral lymph nodes. This might be due to the single-round nature of this vaccine platform.

Sex-linked discrepancies in disease severity have been reported for CCHFV infection in mice, with male animals displaying more severe disease (Hawman et al., 2021). Therefore, we compared humoral and cellular immune responses in vaccinated male and female mice. Other than a modest difference in the kinetics of NP-mediated T cell activation in vaccinated mice, we did not observe any sex-linked differences after VRP vaccination. Enhanced splenocyte cytokine production in female mice compared to male mice after pathogen exposure has been previously reported (Ishikawa and Bigley, 1990; Su and Stevenson, 2000). Estrogen regulation of the IFN-ɣ reporter may be responsible for this (Fox et al., 1991).

Taken together, this study further supports the excellent safety profile of the CCHFV VRP vaccine platform, demonstrated by the absence of detectable innate immune responses. We demonstrate that VRP vaccination results in the development of non-neutralizing NP antibodies with ADCD and ADCP activities suggesting that these may be involved in vaccine-mediated protection against CCHFV. The CCHF VRP robust protection appears to be mediated largely through the NP antigen, though we cannot exclude a role for the GPC antigens, despite the absence of detectable antibodies against GPC components (Gc, Gn, GP38).

## Data availability statement

The original contributions presented in the study are included in the article/**Supplementary Material**, further inquiries can be directed to the corresponding author/s.

## Ethics statement

The animal study was reviewed and approved by Centers for Disease Control and Prevention Institutional Animal Care and Use Committee.

## Author contributions

Conceptualization: CS, EB, EK, FS, and JS. Methodology, investigation, and data analysis: EB, EK, FS, MK, JC-M, JS, SG, SW, TS, and TO’N. Supervision: CS, JM, and SP. Writing, review, and editing: CS, EB, EK, FS, and JS. All authors contributed to the article and approved the submitted version.

## Supplementary material

Supplementary Figure 1 Early innate immune responses after VRP vaccination. **(A)** Cytokine levels in plasma samples obtained from vaccinated mice at the indicated timepoints post vaccination were analyzed by Luminex technology. **(B)** RNA levels of selected immune genes in RNA extracted from whole blood or lymph node samples obtained from vaccinated animals euthanized at the indicated timepoints post vaccination. RNA levels were normalized using a housekeeping gene and samples from mock-vaccinated controls.

Supplementary Figure 2 Immune cells in liver, spleen and peripheral lymph nodes after VRP vaccination. Kinetics of cell populations after CCHFV VRP vaccination. Single-cell suspensions were stained with antibodies listed in **Supplementary Table S1** and analyzed by flow cytometry. Spleen **(A)**, liver **(B)**, and peripheral lymph nodes **(C)** were collected on the indicated timepoints post vaccination. **(D)** Total numbers of B cells found in peripheral lymph nodes or liver of vaccinated animals at the indicated time points post vaccination. #: counting beads were compromised and samples could not be analyzed for cell totals.

Supplementary Table 1 Antibodies used in flow cytometry analyses.

## References

[B1] Appelberg S. John L. Pardi N. Végvári Á Bereczky S. Ahlén G.. (2021). Nucleoside-modified mRNA vaccines protect IFNAR ^-/-^ mice against Crimean Congo hemorrhagic fever virus infection. J. Virol. 96 (3), e0156821. doi: 10.1128/JVI.01568-21 34817199 PMC8826901

[B2] Boshra H. Lorenzo G. Rodriguez F. Brun A. (2011). A DNA vaccine encoding ubiquitinated Rift Valley fever virus nucleoprotein provides consistent immunity and protects IFNAR(-/-) mice upon lethal virus challenge. Vaccine 29 (27), 4469–4475. doi: 10.1016/j.vaccine.2011.04.043 21549790

[B3] Butler A. L. Fallon J. K. Alter G. (2019). A sample-sparing multiplexed ADCP assay. Front. Immunol. 10. doi: 10.3389/fimmu.2019.01851 PMC6700248 31456799

[B4] Clegg J. C. S. Lloyd G. (1987). Vaccinia recombinant expressing lassa-virus internal nucleocapsid protein protects guinea pigs against lassa fever. Lancet 2 (8552), 186–188. doi: 10.1016/s0140-6736(87)90767-7 2885642

[B5] Collins A. M. (2016). IgG subclass co-expression brings harmony to the quartet model of murine IgG function. Immunol. Cell Biol. 94 (10), 949–954. doi: 10.1038/icb.2016.65 27502143

[B6] Dowall S. D. Buttigieg K. R. Findlay-Wilson S. J. D. Rayner E. Pearson G. Miloszewska A.. (2015). A Crimean-Congo hemorrhagic fever (CCHF) viral vaccine expressing nucleoprotein is immunogenic but fails to confer protection against lethal disease. Hum. Vaccines Immunother. 12 (2), 519–527. doi: 10.1080/21645515.2015.1078045 PMC5049717 26309231

[B7] Epstein S. L. Kong W. P. Misplon J. A. Lo C. Y. Tumpey T. M. Xu L.. (2005). Protection against multiple influenza A subtypes by vaccination with highly conserved nucleoprotein. Vaccine 23 (46–47), 5404–5410. doi: 10.1016/j.vaccine.2005.04.047 16011865

[B8] Fischinger S. Fallon J. K. Michell A. R. Broge T. Suscovich T. J. Streeck H.. (2019). A high-throughput, bead-based, antigen-specific assay to assess the ability of antibodies to induce complement activation. J. Immunol. Methods 473, 112630. doi: 10.1016/j.jim.2019.07.002 31301278 PMC6722412

[B9] Fox H. S. Bond B. L. Parslow T. G. (1991). Estrogen regulates the IFN-gamma promoter. J. Immunol. 146 (12), 4362–4367. doi: 10.4049/jimmunol.146.12.4362 1904081

[B10] Goedhals D. Paweska J. T. Burt F. J. (2017). Long-lived CD8 + T cell responses following Crimean-Congo haemorrhagic fever virus infection. PloS Negl. Trop. Dis. 11 (12), e0006149. doi: 10.1371/journal.pntd.0006149 29261651 PMC5752039

[B11] Golden J. W. Shoemaker C. J. Lindquist M. E. Zeng X. Daye S. P. Williams J. A.. (2019). GP38-targeting monoclonal antibodies protect adult mice against lethal Crimean-Congo hemorrhagic fever virus infection. Sci. Adv. 5 (7), eaaw9535. doi: 10.1126/sciadv.aaw9535 31309159 PMC6620094

[B12] Gunn B. M. Lu R. Slein M. D. Ilinykh P. A. Huang K. Atyeo C.. (2021). A Fc-Engineering approach to define functional humoral correlates of immunity against Ebola virus. Immunity 54 (4), 815–828. doi: 10.1016/j.immuni.2021.03.009 33852832 PMC8111768

[B13] Haddock E. Feldmann F. Hawman D. W. Zivcec M. Hanley P. W. Saturday G.. (2018). A cynomolgus macaque model for Crimean – Congo haemorrhagic fever. Nat. Microbiol. 3 (5), 556–562. doi: 10.1038/s41564-018-0141-7 29632370 PMC6717652

[B14] Hawman D. W. Ahlén G. Appelberg K. S. Meade-White K. Hanley P. W. Scott D.. (2020). A DNA-based vaccine protects against Crimean-Congo haemorrhagic fever virus disease in a Cynomolgus macaque model. Nat. Microbiol. doi: 10.1038/s41564-020-00815-6 PMC7854975 33257849

[B15] Hawman D. W. Meade-White K. Leventhal S. Appelberg S. Ahlén G. Nikouyan N.. (2022). Accelerated DNA vaccine regimen provides protection against Crimean-Congo hemorrhagic fever virus challenge in a macaque model. Mol. Ther. 31 (2), 387–397. doi: 10.1016/j.ymthe.2022.09.016 36184852 PMC9931546

[B16] Hawman D. W. Meade-White K. Leventhal S. Carmody A. Haddock E. Hasenkrug K.. (2021). T-cells and interferon gamma are necessary for survival following crimean-congo hemorrhagic fever virus infection in mice. Microorganisms 9 (2), 1–17. doi: 10.3390/microorganisms9020279 PMC7912317 33572859

[B17] Hawman D. W. Meade-White K. Leventhal S. Feldmann F. Okumura A. Smith B.. (2021). Immunocompetent mouse model for Crimean-Congo hemorrhagic fever virus. eLife 10, e63906. doi: 10.7554/eLife.63906 33416494 PMC7811403

[B18] Hawman D. W. Meade-Whitea K. Haddocka E. Habiba R. Scottb TT D. Rosenkeb HF R. (2019). A Crimean-Congo hemorrhagic fever mouse model recapitulating human convalescence 2. J. Virol. 93 (18), e00554–19. doi: 10.1128/JVI.00554-19 31292241 PMC6714788

[B19] Hinkula J. Devignot S. Åkerström S. Karlberg H. Wattrang E. Bereczky S.. (2017). Immunization with DNA plasmids coding for Crimean-Congo hemorrhagic fever virus capsid and envelope proteins and/or virus-like particles. J. Virol. 91 (10), e02076-16. doi: 10.1128/JVI.02076-16 28250124 PMC5411611

[B20] Hu Y. L. Zhang L. Q. Liu X. Q. Ye W. Zhao Y. X. Zhang L.. (2023). Construction and evaluation of DNA vaccine encoding Crimean Congo hemorrhagic fever virus nucleocapsid protein, glycoprotein N-terminal and C-terminal fused with LAMP1. Front. Cell. Infect. Microbiol. 13. doi: 10.3389/fcimb.2023.1121163 PMC10072157 37026060

[B21] Ishikawa R. Bigley N. J. (1990). Sex hormone modulation of interferon (IFN) α/β and γ Production by mouse spleen cell subsets following picornavirus infection. Viral Immunol. 3 (3), 225–236. doi: 10.1089/vim.1990.3.225 2175195

[B22] Kainulainen M. H. Bergeron E. Chatterjee P. Chapman A. P. Lee J. Chida A.. (2021). High-throughput quantitation of SARS-CoV-2 antibodies in a single-dilution homogeneous assay. Sci. Rep. 11 (1), 12330. doi: 10.1038/s41598-021-91300-5 34112850 PMC8192771

[B23] Kang S. M. Compans R. W. (2009). Host responses from innate to adaptive immunity after vaccination: molecular and cellular events. Mol. Cells 27 (1), 5. doi: 10.1007/s10059-009-0015-1 19214429 PMC6280669

[B24] Leventhal S. S. Meade-White K. Rao D. Haddock E. Leung J. Scott D.. (2022). Replicating RNA vaccination elicits an unexpected immune response that efficiently protects mice against lethal Crimean-Congo hemorrhagic fever virus challenge. eBioMedicine 82, 104188. doi: 10.1016/j.ebiom.2022.104188 35907368 PMC9335360

[B25] Matsumura T. Takano T. Takahashi Y. (2022). Immune responses related to the immunogenicity and reactogenicity of COVID-19 mRNA vaccines. Int. Immunol. 35 (5) 213–220. doi: 10.1093/intimm/dxac064 36566501

[B26] Mears M. C. Rodriguez S. E. Schmitz K. S. Padilla A. Biswas S. Cajimat M. N. B.. (2022). Design and evaluation of neutralizing and fusion inhibitory peptides to Crimean-Congo hemorrhagic fever virus. Antiviral Res. 207, 105401. doi: 10.1016/j.antiviral.2022.105401 36049554

[B27] Scholte F. E. M. Spengler J. R. Welch S. R. Harmon J. R. Coleman-McCray J. D. Freitas B. T.. (2019). Single-dose replicon particle vaccine provides complete protection against Crimean-Congo hemorrhagic fever virus in mice. Emerg. Microbes Infect. 8 (1), 575–578. doi: 10.1080/22221751.2019.1601030 30947619 PMC6455139

[B28] Spengler J. R. Welch S. R. Scholte F. E. M. Coleman-McCray J. A. D. Harmon J. R. Nichol S. T.. (2019). Heterologous protection against Crimean-Congo hemorrhagic fever in mice after a single dose of replicon particle vaccine. Antiviral Res. 170 (July), 104573. doi: 10.1016/j.antiviral.2019.104573 31377243 PMC6773275

[B29] Spengler J. R. Welch S. R. Scholte F. E. M. Rodriguez S. E. Harmon J. R. Coleman-McCray J. D.. (2021). Viral replicon particles protect IFNAR–/– mice against lethal Crimean-Congo hemorrhagic fever virus challenge three days after vaccination. Antiviral Res. 191, 105090. doi: 10.1016/j.antiviral.2021.105090 34044061 PMC9250103

[B30] Su Z. Stevenson M. M. (2000). Central Role of Endogenous Gamma Interferon in Protective Immunity against Blood-Stage Plasmodium chabaudi AS Infection. Infect. Immun. 68 (8), 4399–4406. doi: 10.1128/IAI.68.8.4399-4406.2000 10899836 PMC98333

[B31] Tsuda Y. Caposio P. Parkins C. J. Botto S. Messaoudi I. Cicin-Sain L.. (2011). A replicating cytomegalovirus-based vaccine encoding a single ebola virus nucleoprotein CTL epitope confers protection against ebola virus. PloS Negl. Trop. Dis. 5 (8), e1275. doi: 10.1371/journal.pntd.0001275 21858240 PMC3153429

[B32] Welch S. R. Ritter J. M. McElroy A. K. Harmon J. R. Coleman-McCray J. A. D. Scholte F. E. M.. (2019). Fluorescent Crimean-Congo hemorrhagic fever virus illuminates tissue tropism patterns and identifies early mononuclear phagocytic cell targets in IFNAR-/- mice. PloS Pathog. 15 (12), e1008183. doi: 10.1371/journal.ppat.1008183 31790513 PMC6984736

[B33] Welch S. R. Scholte F. E. M. Flint M. Chatterjee P. Nichol S. T. Bergeron É. (2017). Identification of 2′-deoxy-2′-fluorocytidine as a potent inhibitor of Crimean-Congo hemorrhagic fever virus replication using a recombinant fluorescent reporter virus. Antiviral Res. 147 (October), 91–99. doi: 10.1016/j.antiviral.2017.10.008 29024765 PMC8191813

[B34] Wilson J. A. Hart M. K. (2001). Protection from ebola virus mediated by cytotoxic T lymphocytes specific for the viral nucleoprotein. J. Virol. 75 (6), 2660–2664. doi: 10.1128/JVI.75.6.2660-2664.2001 11222689 PMC115890

[B35] Xu X. Ruo S. L. McCormick J. B. Fisher-Hoch S. P. (1992). Immunity to Hantavirus challenge in Meriones unguiculatus induced by vaccinia-vectored viral proteins. Am. J. Trop. Med. Hyg. 47 (4), 397–404. doi: 10.4269/ajtmh.1992.47.397 1359802

[B36] Zivcec M. Guerrero L. I. W. Albariño C. G. Bergeron É Nichol S. T. Spiropoulou C. F. (2017). Identification of broadly neutralizing monoclonal antibodies against Crimean-Congo hemorrhagic fever virus. Antiviral Res. 146, 112–120. doi: 10.1016/j.antiviral.2017.08.014 28842265 PMC7195853

[B37] Zivcec M. Safronetz D. Scott D. P. Robertson S. Feldmann H. (2018). Nucleocapsid protein-based vaccine provides protection in mice against lethal Crimean-Congo hemorrhagic fever virus challenge. PloS Negl. Trop. Dis. 12 (7), e0006628. doi: 10.1371/journal.pntd.0006628 30011277 PMC6062107

