# Supplementary material for: Vaccination with the Crimean-Congo hemorrhagic fever virus viral replicon vaccine induces NP-based T-cell activation and antibodies possessing Fc-mediated effector functions
Source: Front Cell Infect Microbiol. 2023 Aug 21;13:1233148. doi: 10.3389/fcimb.2023.1233148 (PMC10475602; doi:10.3389/fcimb.2023.1233148)
Supplement: Supplementary Table 1 — Antibodies used in flow cytometry analyses. [file Table_1.docx]

**Supplementary Table S1.** Antibodies used in flow cytometry analyses.

| **Marker** | **Fluorophore** | **Clone** | **Vendor** | **Cat. #** |
| --- | --- | --- | --- | --- |
| CD19 | BV650 | 6D5 | BioLegend | 115541 |
| B220 | BV786 | RA3-6B2 | BioLegend | 103245/6 |
| CD45 | PE / PacOr | I3/2.3 | BioLegend | 147711/2 |
| CD40 | PE-Cy7 | 3/23 | BioLegend | 124621/2 |
| MHC II | AF700 | M5/114.15.2 | BioLegend | 107621/2 |
| CD69 | BV421 | H1.2F3 | BioLegend | 104545 |
| CD44 | BV711 | IM7 | BioLegend | 103057 |
| CD8 | PE-Daz594 | 53-6.7 | BioLegend | 100761/2 |
| CD3 | APC | 17A2 | BioLegend | 100209 |
| CD4 | APC-Cy7 | RM4-5 | BioLegend | 100525/6 |

**Supplementary Methods**

Protein expression and purification

For the expression of all CCHFV proteins, Kosovo Hoti strain sequences were used (S segment #DQ133507.1, M segment #EU037902.1). The NP sequence was optimized for bacterial expression and cloned into pET28a by Twist Bioscience. The expression plasmid contained an N-terminal His Tag followed by GST and an HRV 3C protease cleavage site. The construct was transformed into *E. coli* BL21 (DE3) strain (ECO114; ThermoFisher) and a bacterial culture was grown in Luria broth with kanamycin. The culture was induced with 1 mM isopropyl β-D-1-thiogalactopyranoside (IPTG) when optical density was between 0.4-0.6. Following induction, the culture was transferred to 16°C for overnight incubation. Cells were harvested by centrifugation, resuspended in lysis buffer (500 mM NaCl, 20 mM Tris-Cl [pH 7], 0.1% Triton-X, 5% glycerol, 1 mM MgCl_2_, 25 U/ml benzonase), and sonicated. The cleared lysates were filtered through 0.2-micron polyethersulfone (PES) membranes and loaded onto HisTrap Excel columns (17371206, Cytiva) for immobilized metal affinity chromatography (IMAC). Following His purification, N-terminal His-GST was cleaved with HRV-3C protease cleavage enzyme (3CC-N3133, Acro Biosystems).

The CCHFV Gn (Gn-HRV 3C protease cleavage site-His Tag-Twin-Strep Tag), Gc (Mucin-GP38-Furin cleavage site-Gc-HRV 3C protease cleavage site-His Tag-Twin-Strep Tag-), and GP38 (P85-HRV 3C cleavage site-His Tag-Twin Strep Tag) sequences were cloned into pTWIST (for Gn) or pEEV(Kainulainen et al., 2021) (for Gc and GP38) plasmids by Twist Bioscience. The proteins were expressed in Expi293F cells (A14527; ThermoFisher) growing in Expi293 Expression medium after transient transfection using FectoPro transfection reagent (101000007; Polypus). For the constructs with furin cleavage sites, plasmids were co-transfected with furin plasmid at a 4:1 ratio (CCHFV protein expression plasmid:furin plasmid). Cells were harvested 4-6 days post transfection, supernatants were filtered through 0.2-micron PES membranes and purified by IMAC using HisTrap Excel columns with a final 500 mM NaCl concentration. Proteins were further purified using size exclusion chromatography (HiLoad 16/600 Superdex 200 pg, GR28-9893-35), quantified, and stored at -80°C. All expression and purification steps were confirmed by polyacrylamide gel electrophoresis.

ELISA

Immulon 2HB plates were coated with 50 ng antigen in PBS and incubated overnight at 4°C. Wells were washed with PBS-T (0.1% Tween-20 in PBS) and blocked with blocking buffer (5% [w/v] non-fat dry milk in PBS-T) for 1 h at room temperature (RT). Following blocking, buffer was decanted and 100 μl of mouse plasma samples diluted in blocking buffer was added to the wells in duplicates. For IgG and IgM ELISA, plasma samples were used at 1:1000 and 1:500 dilutions, respectively. After 1h incubation at RT, wells were washed and anti-mouse IgG HRP (1:3000) or anti-mouse IgM HRP (1:1000) was added to the wells, and incubated 1h at RT. Wells were washed, and TMB Ultra ELISA substrate (ThermoFisher) was added and incubated 10 min at RT. The reaction was stopped using ELISA stop solution (ThermoFisher) and optical density (OD) was read at 450 nm. Antibody activity units were determined based on calibrator curves generated using mouse IgG and IgM standards. Antibody concentrations in samples were determined by interpolating the concentrations of the standards that corresponded to the absorbance value of the sample.

IgG avidity of NP-specific antibodies was determined by treating plasma samples with 1M NH_4_SCN prior to ELISA analysis as described above. IgG avidity index was calculated by dividing the mean OD of NH_4_SCN-treated plasma by the mean OD of PBS-treated plasma, and multiplying this number by 100 to obtain a percentage.

Flow analysis

Spleen, liver, and peripheral lymph nodes were obtained from euthanized animals. Single-cell suspensions were obtained from livers and spleens by mashing tissue through a 70 µm mesh filter. Red blood cells in spleen lysates were lysed using RBC lysis buffer (Roche). Immune cells were isolated from liver lysates using Histopaque and washed with RPMI before further processing. Several peripheral lymph nodes obtained from each animal were combined and processed as one sample. Lymph node capsules were broken open using a plastic mortar in an Eppendorf tube and passed through a 70 µm mesh filter to obtain single-cell suspensions, followed by a wash with RPMI.

Cells were incubated with mouse Fc block diluted in PBS and subsequently incubated with surface stains (Supplementary table S1) for 30 min on ice. Cells were washed and resuspended in Cytofix/Cytoperm (BD) for 20 min on ice. Cell viability was determined using Tonbo Ghost Dye Violet 510. After additional washes, cells were resuspended in flow buffer (PBS + 1% heat-inactivated FBS) and analyzed on a Stratedigm S1000EXi.

Antibody-dependent complement deposition (ADCD)

The ADCD assay was adapted from Fischinger et al (Fischinger et al., 2019). Recombinant CCHFV NP (Hoti strain, produced as described above) was biotinylated (21435, EZ-Link™ Sulfo-NHS-LC-Biotinylation Kit) and coupled to 1.0 μm fluorescent red neutravidin microspheres (ThermoFisher F8775). Excess antigen was washed away with PBS containing 5% BSA. Antigen-coated beads were incubated with mouse plasma (2 h at 37°C) and unbound antibodies were washed away with PBS. Guinea pig complement (Cedarlane, CL4051) diluted in gelatin veronal buffer (CompTech B102) was added and incubated 15 min at 37°C. Immune complexes were washed with 15 mM EDTA in PBS and incubated 15 min at room temperature with FITC-conjugated goat IgG fraction to guinea pig complement C3 (MP Biomedicals, 0855385). Unbound antibody was washed away with PBS and immune complexes were analyzed on a Guava Cytometer. Fold ADCD activation was calculated using naïve mouse plasma.

Antibody-dependent cellular phagocytosis (ADCP)

The ADCP assay was adapted from Butler et al (Butler et al., 2019). Immune complexes were formed as described for ADCD, with the exception that biotinylated antigen was coupled to 1.0 μm fluorescent green neutravidin microspheres (ThermoFisher F8776). Immune complexes were incubated overnight at 37°C with 1E+04 THP-1 cells per well. The next day cells were washed and analyzed on a Guava Cytometer. Phagocytic score was calculated by multiplying the percentage of bead-positive cells with the overall median fluorescence intensity.

**References**

Butler, A.L., Fallon, J.K., Alter, G., 2019. A Sample-Sparing Multiplexed ADCP Assay. Frontiers in Immunology 10.

Fischinger, S., Fallon, J.K., Michell, A.R., Broge, T., Suscovich, T.J., Streeck, H., Alter, G., 2019. A high-throughput, bead-based, antigen-specific assay to assess the ability of antibodies to induce complement activation. Journal of Immunological Methods 473, 112630. https://doi.org/10.1016/j.jim.2019.07.002

Kainulainen, M.H., Bergeron, E., Chatterjee, P., Chapman, A.P., Lee, J., Chida, A., Tang, X., Wharton, R.E., Mercer, K.B., Petway, M., Jenks, H.M., Flietstra, T.D., Schuh, A.J., Satheshkumar, P.S., Chaitram, J.M., Owen, S.M., McMullan, L.K., Flint, M., Finn, M.G., Goldstein, J.M., Montgomery, J.M., Spiropoulou, C.F., 2021. High-throughput quantitation of SARS-CoV-2 antibodies in a single-dilution homogeneous assay. Sci Rep 11, 12330. https://doi.org/10.1038/s41598-021-91300-5
